# Systemic therapy in metastatic renal cell carcinoma

**DOI:** 10.1007/s00345-016-1868-5

**Published:** 2016-06-09

**Authors:** Jens Bedke, Thomas Gauler, Viktor Grünwald, Axel Hegele, Edwin Herrmann, Stefan Hinz, Jan Janssen, Stephan Schmitz, Martin Schostak, Hans Tesch, Stefan Zastrow, Kurt Miller

**Affiliations:** 1 Department of Urology, Eberhard Karls University Tübingen, Hoppe-Seyler-Strasse 3, 72076 Tübingen, Germany; 2 Department of Radiation Oncology, University of Essen, Essen, Germany; 3 Department of Hematology and Oncology, Medical School Hannover, Hannover, Germany; 4 Department of Urology and Pediatric Urology, University of Marburg, Marburg, Germany; 5 Department of Urology, University of Münster, Münster, Germany; 6 Department of Urology, Charité Universitaetsmedizin Berlin, Berlin, Germany; 7 Onkologie Westerstede, Westerstede, Germany; 8 Gemeinschaftspraxis für Onkologie und Hämatologie, Köln, Germany; 9 Department of Urology, University of Magdeburg, Magdeburg, Germany; 10 Onkologie Bethanien, Frankfurt am Main, Germany; 11 Department of Urology, Technical University of Dresden, Dresden, Germany

**Keywords:** Renal cell carcinoma, Systemic treatment, Targeted therapy, Tyrosine kinase inhibitor mTOR inhibition, Checkpoint inhibitor, Sequence

## Abstract

**Purpose:**

Current systemic treatment of targeted therapies, namely the vascular endothelial growth factor-antibody (VEGF-AB), VEGF receptor tyrosine kinase inhibitor (TKI) and mammalian target of rapamycin (mTOR) inhibitors, have improved progression-free survival and replaced non-specific immunotherapy with cytokines in metastatic renal cell carcinoma (mRCC).

**Methods:**

A panel of experts convened to review currently available phase 3 data for mRCC treatment of approved agents, in addition to available EAU guideline data for a collaborative review as the plurality of substances offers different options of first-, second- and third-line treatment with potential sequencing.

**Results:**

Sunitinib and pazopanib are approved treatments in first-line therapy for patients with favorable- or intermediate-risk clear cell RCC (ccRCC). Temsirolimus has proven benefit over interferon-alfa (IFN-α) in patients with non-clear cell RCC (non-ccRCC). In the second-line treatment TKIs or mTOR inhibitors are treatment choices. Therapy options after TKI failure consist of everolimus and axitinib. Available third-line options consist of everolimus and sorafenib. Recently, nivolumab, a programmed death-1 (PD1) checkpoint inhibitor, improved overall survival benefit compared to everolimus after failure of one or two VEGFR-targeted therapies, which is likely to become the first established checkpoint inhibitor in mRCC. Data for the sequencing of agents remain limited.

**Conclusions:**

Despite the high level of evidence for first and second-line treatment in mRCC, data for third-line therapy are limited. Possible sequences include TKI-mTOR-TKI or TKI–TKI-mTOR with the upcoming checkpoint inhibitors in perspective, which might settle a new standard of care after previous TKI therapy.

## Introduction

Kidney cancer accounts for about 2–3 % of all cancers in the world each year and is the third-most common urological tumor. The most common type of renal tumors in adults is renal cell carcinoma (RCC), which represents approximately 90–95 % of all cases [1, 2].

**Fig. 1:**
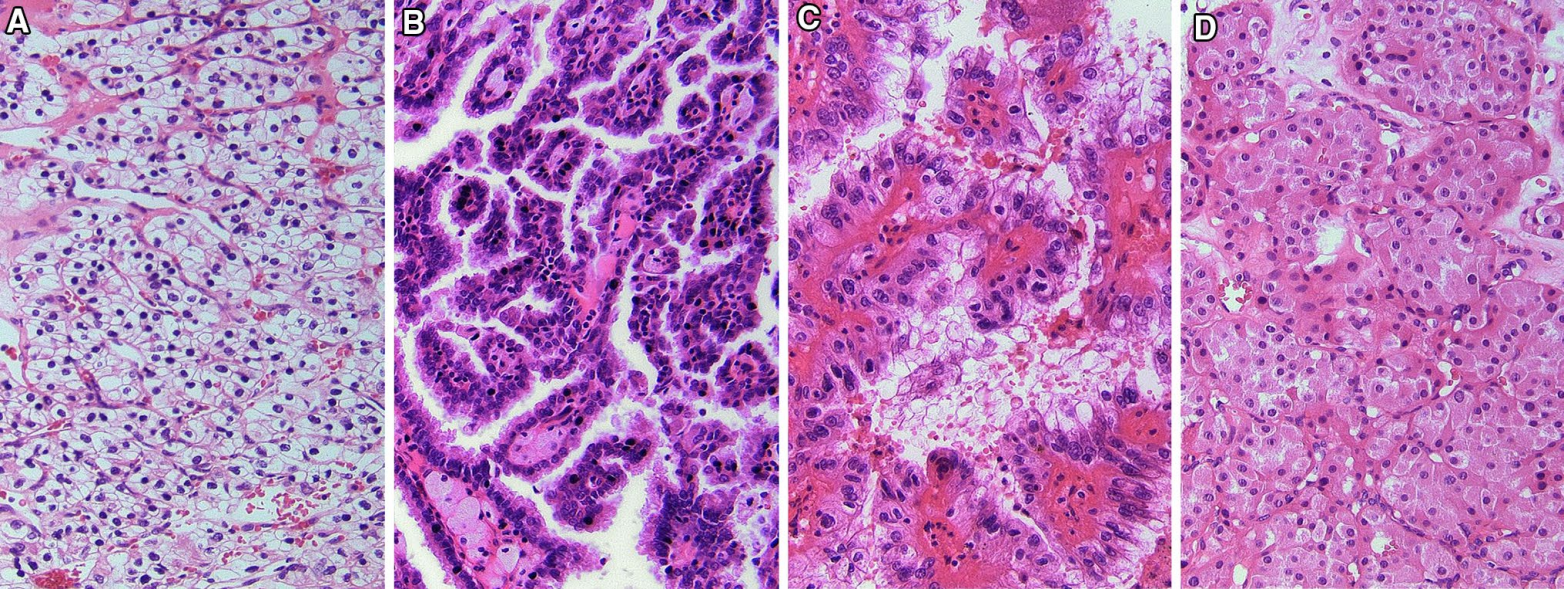
Histological subtypes of renal cell carcinoma. **a** Clear cell RCC (×20). **b** Papillary RCC Type I (×20). **c** Papillary RCC Type II (×20). **d** Chromophobe RCC (×40)

RCC includes different entities, with the most common histological subtype being clear cell (ccRCC) of about 75–80 % (Fig. 1). All other subtypes are summarized as non-clear cell RCC, which include papillary (papRCC), chromophobe RCC (chRCC) and various other entities [3]. The different subtypes are characterized by their distinct molecular patterns, which reflect pathway alterations leading to the tumor growth. These alterations arise from inherited genetic disorders, which underlie a specific syndrome, or more often from sporadic non-hereditary mutations [4, 5].

CcRCC is characterized by the inactivation of the von Hippel-Lindau (VHL) tumor suppressor gene by mutation and promoter hypermethylation. The discovery of the VHL signaling pathway and its implications are the backbone for modern molecular targeted therapies of metastatic renal cell carcinoma (mRCC) with VEGF (vascular endothelial growth factor) and mTOR (mammalian target of rapamycin) signaling pathway inhibitors [6, 7].

### Therapy options for mRCC

The multi-targeted tyrosine kinase inhibitors (TKI) axitinib, pazopanib, sorafenib and sunitinib inhibit signaling cascades activated by peripheral membrane receptor tyrosine kinases (RTK) like the VEGF receptor (VEGFR), which stimulates proliferation, cell survival and angiogenesis.

The intracellular signaling PI3 K/AKT/mTOR pathway, important in regulating the cell cycle, is one of the most deregulated pathways; everolimus and temsirolimus interfere directly with it by acting on mTOR, reducing the activity of the effector molecules S6K1 and 4EBP1, and increasing the synthesis of cycle proteins like HIF-1α. As a consequence, they inhibit cell proliferation, growth and survival, and interrupt the cell cycle in the G1-phase [8–10].

The monoclonal antibody bevacizumab binds the growth factor VEGF-A in the extracellular space, inhibiting it from binding to its associated receptor VEGFR. Consequently, the signaling pathways activated by VEGF are interrupted at the surface of the cell [9].

Non-specific immunotherapy with the cytokines interleukin-2 (Il-2) and interferon-alfa (IFN-α) had been the standard treatment option for mRCC in the past. These drugs were established as the first effective immunotherapy and were used in combinations as well as monotherapy. Il-2 has effectively fallen from routine use since the new targeted therapies demonstrated higher efficacy in overall survival (OS) in numerous clinical trials and proved to be effective in clinical practice.

The introduction of targeted therapies, namely the VEGF-TKI and mTOR inhibitors, has improved progression-free survival (PFS) and expanded the treatment options. The aim of this paper is to review currently available phase 3 data for mRCC treatment as the plurality of approved agents offer different options of first-, second- and third-line treatment with potential sequencing.

## Materials and methods

A panel of experts convened to review currently available phase 3 data for mRCC treatment of approved agents to perform a collaborative review. Evidence acquisition is based on the search results of the EAU guidelines update from 2014 as a basis [1] and moved from there on by adding the most recent publications of comparative randomized and a few non-randomized studies. Level of evidence is given according to a classification system modified from the Oxford Centre for Evidence-Based Medicine Levels of Evidence as used in the EAU guidelines [1, 11].

## Results

### First-line treatment

**Fig. 2:**
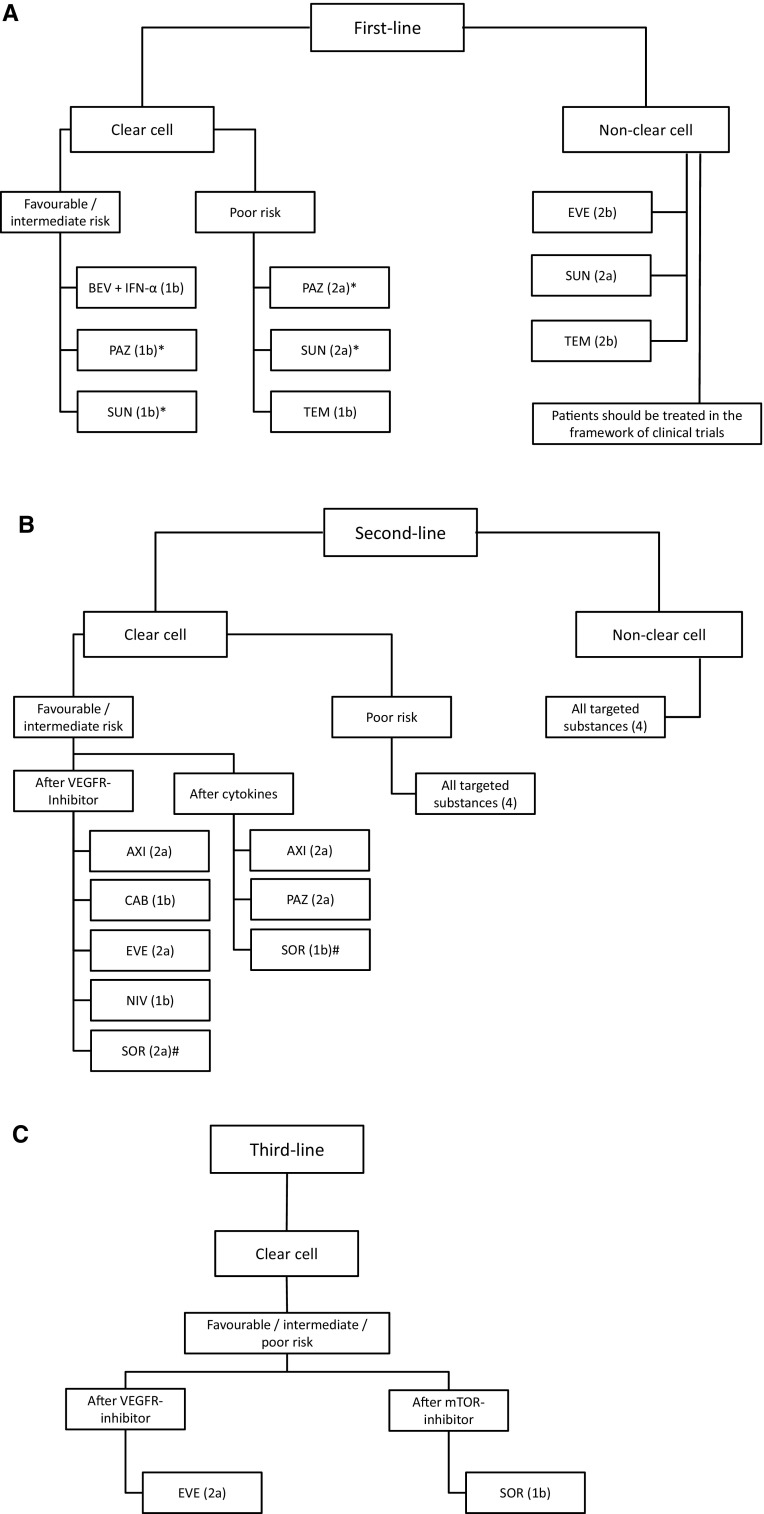
**a**–**c** Flow charts of the potential therapeutic options for the first-, second- and third-line treatment of mRCC (levels of evidence in *brackets*). *BEV* bevacizumab, *IFN-α* Interferon alfa, *EVE* everolimus, *PAZ* pazopanib, *SUN* sunitinib, *TEM* temsirolimus, *AXI* axitinib, *SOR* sorafenib, *VEGFR* vascular endothelial growth factor receptor, *mTOR* mammalian target of rapamycin, *NIV* nivolumab, *CAB* cabozantinib. *Level of evidence for pazopanib and sorafenib in poor risk patients is 2a as data are based on a subgroup analysis. Level of evidence for the entire cohort is 1b. #Sorafenib was inferior to axitinib in a RCT in terms of PFS, but not different in OS [28]

The optimal therapy for patients with ccRCC is generally chosen after the stratification according to the MSKCC (Memorial Sloan-Kettering Cancer Center) or IMDC (International Metastatic Renal Cell Carcinoma Database Consortium) criteria [12, 13]. The vast majority of patients have a favorable or intermediate prognostic risk; a number of different treatment options approved for first-line therapy with equivalent levels of evidence are available for these patients (Fig. 2a).

For patients with an unfavorable prognosis, temsirolimus showed a survival benefit when compared to IFN-α; therefore, this mTOR inhibitor is regarded as a standard for this subgroup of patients [1, 14]. Pazopanib and Sunitinib can be used as an alternative treatment [1].

For patients with non-ccRCC, the prognosis is considerably worse [15]; the available scientific data are scarce as phase 3 studies that could have defined a standard are missing. Data from phase 2 studies and expanded access programs suggest the efficacy of a few agents (temsirolimus, everolimus, sunitinib and sorafenib) [1]. The EAU guidelines recommend these patients preferably to be treated in a clinical trial. If there is no study available, non-ccRCC patients can be treated similarly to ccRCC patients, temsirolimus, everolimus or the VEFGR-targeted therapies (sunitinib or sorafenib) could be considered as treatment options [1].

#### TKI sunitinib and pazopanib

The cytokines Il-2 and IFN-α alone, or in combination with 5-fluorouracil, dominated the systemic therapy of mRCC for many years, until the introduction of targeted agents like sunitinib or pazopanib led to major improvements in efficacy. Sunitinib was the first of these novel agents, almost doubling the PFS of patients with mRCC compared to IFN-α (HR 0.42; CI 95 % 0.32–0.54; *p* < 0.001) [16]. A significant benefit in OS could not be shown (26.4 vs. 21.8 months; HR 0.821; CI 95 % 0.673–1.001; *p* = 0.051), probably due to the survival endpoint being confounded by crossover to sunitinib [17]. Pazopanib showed similar efficacy to sunitinib in a placebo-controlled randomized phase 3 trial, with a median PFS of 11.1 months compared to 2.8 months in the placebo arm (HR 0.40; CI 95 % 0.27–0.6) [18]. A significant difference in OS could also not be demonstrated (median OS 22.9 vs. 20.5 months, HR 0.91; CI 95 % 0.71–1.16; *p* = 0.224)—probably also because of crossover from placebo to pazopanib in more than 80 % of patients [19]. The efficacy and safety of both agents were discussed widely, but they were not compared with each other in an unblinded controlled trial until the COMPARZ study, the first comparative trial of two TKIs in the first-line treatment of mRCC. It showed non-inferiority of pazopanib compared to sunitinib in PFS (8.4 vs. 9.5 months; HR 1.05; CI 95 % 0.90–1.20; *p* < 0.05) [20]. A similar OS outcome supported the findings of the primary analysis of PFS and set a new benchmark for expected survival in mRCC (28.4 vs. 29.3 months; HR 0.91; CI 95 % 0.79–1.06; *p* = 0.275) [21]. While the efficacy of both agents is similar, differences in the toxicity profiles were noticed: The frequency of fatigue, hand-foot syndrome, and thrombocytopenia was higher with sunitinib; frequency of weight loss, alopecia and liver function abnormalities were higher with pazopanib [21].

#### Combination of bevacizumab plus IFN-α

The combination of bevacizumab with IFN-α as first-line treatment in patients with mRCC was investigated in the AVOREN study with a significant improvement in PFS compared to IFN-α alone. The median PFS in the combination was 10.2 months compared to 5.4 in the control group (HR 0.63; CI 95 % 0.52–0.75; *p* = 0.0001) [22]. Nevertheless, the difference in median OS, the primary endpoint—23.3 months with bevacizumab plus IFN-α compared to 21.3 months with IFN-α plus placebo—was not significant (un-stratified HR 0.91; CI 95 % 0.76–1.10; *p* = 0.336). The fact that a statistically significant benefit in OS could not be observed is possibly the result of confounding factors, like crossover of patients in the IFN-α to bevacizumab before progression or multiple lines of post-study therapy (this was the case for 55–63 % of patients) [23].

#### mTOR inhibitor temsirolimus

In a phase 3 trial, which later supported the marketing approval of temsirolimus, 626 patients with previously untreated, clear cell (80 %) and non-clear cell (20 %) mRCC with mostly poor prognosis (72 %) were randomly assigned to receive temsirolimus, IFN-α, or the combination therapy of temsirolimus plus IFN-α [24]. The primary endpoint was OS in the temsirolimus group and the combination therapy group, in comparison with the IFN-α group. It became apparent that the OS of patients in the temsirolimus group was significantly longer than that in the IFN-α group alone (10.9 vs. 7.4 months; HR 0.73; CI 95 % 0.58–0.92). A survival benefit could not be shown for the combination therapy in comparison with the IFN-α monotherapy (median OS 8.4 vs. 7.3 months; HR 0.96; CI 95 % 0.76–12.0). The differences in PFS were also not statistically significant (5.5 months for temsirolimus, 4.7 for the combination therapy and 3.1 for IFN-α alone).

### Second-line treatment

For the second-line treatment of mRCC, there are several targeted agents available, like TKI or mTOR inhibitors, with different levels of evidence and grades of recommendation (Fig. 2b). Axitinib, everolimus and sorafenib are available for patients who have failed VEGFR-targeted therapy. Axitinib, pazopanib and sorafenib can be given after failure of prior cytokines, although this is becoming rather uncommon in current clinical practice. For patients with clear cell histology and unfavorable risk, and those with non-clear cell histology, the EAU guidelines stipulate a treatment with targeted agents with a level of evidence 4 [1].

#### TKI axitinib and sorafenib and mTOR inhibitor everolimus

The RECORD-1 study investigated everolimus versus placebo as second- or third-line therapy after failure of one or two VEGFR-targeted therapies in patients with mRCC. An advantage in PFS could be demonstrated for the treatment with everolimus [25, 26]. The median PFS in patients pre-treated with a TKI was 4.9 months compared to 1.9 with placebo (HR 0.33; CI 95 % 0.24–0.43; *p* < 0.001). Axitinib versus sorafenib as a second-line therapy was tested in the AXIS study, which resulted in a significantly longer PFS for axitinib. Patients who had progressed under an approved systemic therapy (containing sunitinib, bevacizumab plus IFN-α, temsirolimus or cytokines) had a median PFS of 4.8 months under axitinib, in comparison with 3.4 months under sorafenib (HR 0.741; CI 95 % 0.573–0.958) [27]. Nevertheless, neither axitinib nor everolimus demonstrated an advantage in terms of OS; the difference in OS between everolimus and placebo was not significant (14.8 vs. 14.4 months; HR 0.87; CI 95 % 0.65–1.17) [26]. This was also due to the trial design, which allowed a crossover from placebo to everolimus after disease progression—80 % of patients in the placebo arm made use of this possibility. In the AXIS study there was no difference in the secondary endpoint of OS between the two study arms. Patients treated with axitinib had an OS of 15.2 months compared to 16.5 months for patients treated with sorafenib (HR 0.997; CI 95 % 0.782–1.27) [28]. Again this outcome could be because the patients received numerous treatments in the subsequent therapy lines.

#### Nivolumab

Nivolumab, a programmed death 1 (PD1)-antibody, interferes with the immune system as a checkpoint inhibitor by solving the break on the cytotoxic T cells. Showing promising activity in heavily pre-treated mRCC patients, it was tested in a large phase 3 trial of 822 patients with metastatic or advanced RCC (CheckMate 025) [29, 30]. Patients with 1 or 2 prior anti-angiogenic therapies, which is defined as prior VEGFR-targeted or anti-VEGF-antibody therapy, were randomized to receive either nivolumab (3 mg/kg every 2 weeks) or everolimus (10 mg p.o daily). The primary study endpoint OS was reached with a significant benefit for nivolumab (25.0 vs. 19.6 months, HR 0.73; *p* = 0.002). This is the first study in the area of targeted treatments in mRCC, which showed a clear survival benefit, a better response rate (25 vs. 5 %; odds ratio 5.98 [95 % CI, 3.68–9.72]; *p* < 0.001), a statistically better quality of life and less toxicity (19 vs. 37 % grade III/IV adverse events).

#### Cabozantinib

Cabozantinib, a TKI that targets MET and VEGFR-2, is another new substance that has been tested in a phase 3 study (METEOR) as a second-line treatment for patients with mRCC that had received at least one TKI treatment [31]. Patients were randomized to 60 mg of cabozantinib or 10 mg of everolimus, a crossover was not allowed. The primary endpoint of the study was PFS. Median PFS was 7.4 months with cabozantinib versus 3.8 months with everolimus (HR 0.58; 95 % CI, 0.45–0.75; *p* < 0.001); an interim analysis of the OS data showed a survival benefit of 33 % for cabozantinib (HR 0.67; 95 % CI 0.51–0.89; *p* = 0.005) [31].

### Third-line treatment

Clinical trials in the third-line setting are limited to the GOLD and the RECORD-1 studies. The recommendation that might be deduced from the results of these studies is that a third-line therapy should be chosen depending on the previous treatment sequence in the first- and second-line, it only applies to ccRCC (Fig. 2c). Sorafenib can be recommended after a sequence of TKI-mTOR inhibitor (level of evidence 1b), based on the data of the GOLD study [32]. Treatment with the mTOR inhibitor everolimus can be given after the sequence TKI–TKI, this recommendation being based on a subgroup analysis of the RECORD-1 study [33].

#### Everolimus and sorafenib

Although the GOLD trial failed to demonstrate superior efficacy of dovitinib, a tyrosine kinase inhibitor that targets VEGF and FGF receptors, over sorafenib in patients who had progressed on prior VEGFR and mTOR inhibitor therapies, the results suggest the efficacy and safety of sorafenib in the third-line setting with a median PFS of 3.7 versus 3.6 months in the placebo arms (HR 0.86; CI 95 % 0.72–1.04; *p* = 0.063) [32].

A subset analysis of the RECORD-1 study, which compared everolimus plus best supportive care (BSC) versus placebo plus BSC in patients who had received one or several previous treatments, showed a median PFS of 4.0 months after pre-treatment with two TKIs. The median PFS was 2.2 months longer than under placebo, which demonstrated the activity of everolimus in third-line therapy (HR 0.32; CI 95 % 0.19–0.45) [33].

## Discussion and conclusions

For first- and second-line therapy, there is now a growing evidence to guide the selection of the appropriate treatment; especially as comparative studies between targeted therapies are appearing. For the third-line, the evidence is still somewhat limited as is the information on the best sequential therapy.

In first-line therapy, sunitinib and pazopanib are the treatments of choice for patients with favorable or intermediate prognostic risk features and ccRCC. Pazopanib was not inferior to sunitinib in a phase 3 study and sunitinib proved to be superior to IFN-α [16, 18]. Both agents have comparable efficacy and should be chosen at the physician’s discretion depending on treatment tolerance and patient preference. Bevacizumab combined with IFN-α represents an effective alternative for the first-line as well, and is particularly relevant for younger patients with a favorable risk. Essential for the choice of the targeted therapy for tumors with clear cell histology is a classification according to the risk models MSKCC or IMDC. In patients with a poor risk score, temsirolimus is the one valid therapy option. In this setting, temsirolimus showed a prolonged OS compared to IFN-α. In patients with non-ccRCC and/or poor prognostic risk, temsirolimus represents the standard of care supported by phase 3 data. Alternatives include sunitinib and everolimus [1].

Real-life data based on prospective registry data in Germany underline that the recommended guideline therapy is followed in clinical practice. Pazopanib and sunitinib are the most commonly used drugs in first-line therapy of mRCC in Germany, followed by temsirolimus; other agents like bevacizumab, IFN-α or sorafenib play a minor role according to a cancer registry for advanced RCC conducted by the clinical research organization iOMEDICO (iOMEDICO AG, Freiburg, Germany), as per April 2014 [34]. The use of sunitinib in this indication has decreased continuously over the last 7 years, while the use of pazopanib has increased in the meantime.

In second-line therapy, it remains unclear whether TKI or mTOR inhibitors are the better choice. Therapy options after TKI failure consist of everolimus and axitinib and—with a lower level of evidence—sorafenib. Axitinib has proven efficacy and superiority in terms of PFS in comparison with sorafenib after failure of the first systemic therapy. Everolimus prolongs PFS in comparison with placebo in patients who have previously failed or are intolerant to first-line VEGFR-TKI therapy. However, neither axitinib nor everolimus demonstrated an advantage in terms of OS, and there is no direct comparison between the two substances. Not surprisingly, everolimus and axitinib are currently the most commonly used agents for second-line therapy of mRCC in Germany according the iOMEDICO cancer registry [34].

Despite the limited number of phase 3 trials in this setting, it is widely accepted that patients who retain a good performance status may still benefit from a third line of therapy. Available therapy options are everolimus after two previous lines of TKI and sorafenib after a first-line of TKI followed by an mTOR inhibitor. The median PFS reached in the third line are comparable to that in the second-line [33]. In current medical care of mRCC, almost half of the patients who received a second-line treatment are treated in a third-line (25 % of all patients treated with a systemic therapy) and one-fifth get a fourth-line (10 % of patients with a systemic therapy) [34].

### Sequencing in first- and second-line therapy

Seven new targeted therapies (axitinib, bevacizumab + IFN-α, everolimus, pazopanib, sorafenib, sunitinib and temsirolimus) and one checkpoint inhibitor (nivolumab) with proven efficacy have been approved since 2005 for the treatment of mRCC, and real-world data reflect their use in clinical practice. But what is the optimal sequence of these agents?

There is little evidence available on the optimum sequence of these agents in first or second-line therapy; consequently, the EAU guidelines give no firm recommendation on the best sequence for targeted therapy.

RECORD-3 was the first randomized phase 2 study to prospectively compare the sequence of everolimus followed by sunitinib to the sequence of sunitinib followed by everolimus. While the most important prognostic patient characteristics were equally distributed between the arms, there was no significant difference in median combined PFS, a secondary endpoint of the study, between the sequence sunitinib–everolimus (22.2 months) and that of everolimus–sunitinib (21.7 months; HR 1.2; CI 95 % 0.9–1.6) [35]. The censoring rates were high in both arms: 56 % for everolimus–sunitinib and 57 % for sunitinib–everolimus; mainly because of patients who did not cross over to a second-line therapy within the protocol study period. Patients who never received a per-protocol second-line therapy were also censored. Although the censoring could have impacted the Kaplan–Meier and HR estimates, the combined median PFS of 22.2 months was an endpoint that had not been established previously in a prospective trial, and will serve as a benchmark for future trials in sequential therapy. The results of the study confirmed first-line sunitinib followed by everolimus at progression as one possible sequence for the treatment of mRCC.

Another trial examining sequential therapy was SWITCH-I, a randomized phase 3 study, which evaluated the efficacy and safety of sorafenib followed by sunitinib versus sunitinib followed by sorafenib. Based on retrospective data, it was hypothesized that sorafenib–sunitinib might be a statistically superior sequence; however, there was no significant difference in total PFS between both arms. The median total PFS was 12.5 months in the sorafenib–sunitinib arm and 14.9 months in the sunitinib–sorafenib arm (HR 1.01; CI 95 % < 1.27; *p* = 0.54) [36]. The OS analysis of the SWITCH-I study showed no superiority of either of the two sequences (median OS 31.5 months for sorafenib–sunitinib vs. 30.2 months for sunitinib–sorafenib; HR 1.00; CI 95 % < 1.3) [36]. A direct comparison of the combined PFS in the RECORD-3 and the total PFS in the SWITCH study is statistically not valid due to different trial settings and different methodology of the endpoints. The AE profiles differed between the individual study medications of both trials, but were generally consistent with previously reported safety profiles for these agents in patients with mRCC [37]. Preliminary results of the RECORD-3 study demonstrated that rates of grade 3 and 4 AEs were higher with a first-line TKI—in this case sunitinib—than with a second-line TKI [35]. This finding seems to be consistent with the results of the SWITCH-I study, and with previous data, showing that grade 3 and 4 AEs tend to decrease in the course of TKI therapy [36, 38].

In summary, treatment options for mRCC have expanded enormously, since the introduction of targeted therapies, and have significantly extended the survival of mRCC patients—OS can be prolonged by up to 32 months by sequencing different approved targeted drugs [20, 23, 39]—but the availability of numerous alternative therapies creates a challenge how to select the optimal treatment protocol.

The molecular biology underlying cancer growth and control is a field of considerable ongoing research. In mRCC, the research has concentrated on TKI and mTOR inhibitors, which have improved patient survival in general, but with a limited prognosis. Therapeutics, which yield a longer lasting response, are warranted, especially those who would provoke long-term response or complete remission as observed with non-specific immunotherapy with the cytokines Il-2 and IFN-α. Another exploratory way for the future to stimulate the immune system is the vaccination with tumor-associated peptides (TUMAP), which aims at activating specific T lymphocytes against tumor tissues. The vaccine approach will need further exploration as latest data of peptide vaccination in the combination with sunitinib in mRCC as a first-line treatment has failed [40, 41].

Recently, a deeper understanding of the underlying immunology of T cell activation led to the development of immune checkpoint inhibitors. These are monoclonal antibodies, which inhibit the PD-1 (CD279) and CTLA-4 (CD152) axis, thereby releasing the inhibition of T cell activation [42]. This approach has shown excellent results with other tumor entities (melanoma, lung cancer) with a subgroup of patients experiencing long-term complete or partial remissions. Phase 3 results in RCC were presented and published for the first time in September 2015. Based on this data, nivolumab will probably drive the second- and third-line treatment of mRCC after failure of a VEGF-targeted therapy [43]. Cabozantinib also shows a promising efficacy in patients that had progressed under TKI. The final results of the OS data remain to be seen, but it seems that a survival benefit yet unequaled in second-line therapy could be achieved [43].
